# Response to Biologic Disease-Modifying Anti-Rheumatic Drugs after Discontinuation of Anti-Tumor Necrosis Factor Alpha Agents for Rheumatoid Arthritis

**DOI:** 10.1007/s40744-014-0002-7

**Published:** 2014-09-23

**Authors:** Martin J. Bergman, Eric P. Elkin, Sarika Ogale, Tripthi Kamath, Max I. Hamburger

**Affiliations:** 1 Taylor Hospital, 175 East Chester Pike, Ridley Park, PA 19078 USA; 2 ICON Clinical Research, 456 Montgomery Street, Suite 2200, San Francisco, CA 94104 USA; 3 Genentech, Inc., 1 DNA Way, South San Francisco, CA 94080 USA; 4 Rheumatology Associates of Long Island, 1895 Walt Whitman Road, Melville, NY 11747 USA

**Keywords:** Anti-rheumatic agents, Arthritis, rheumatoid, Biological therapy, Treatment failure, Tumor necrosis factor alpha

## Abstract

**Introduction:**

The aim of this study was to compare the response between subsequent use of anti-tumor necrosis factor α (anti-TNF) agents and biologic disease-modifying anti-rheumatic drugs (bDMARD) with other mechanism of action (MOA) in rheumatoid arthritis (RA) patients with history of anti-TNF treatment as their first bDMARD.

**Methods:**

A retrospective chart review was conducted at eight community-based rheumatology practices in the United States in 2012. Routine Assessment of Patient Index Data 3 (RAPID3) response was measured by comparing baseline and 6-month scores. Poor response was defined as decrease <1.8 points, follow-up score >12, or treatment discontinuation before 6 months. Percentages of patients with good and good or moderate RAPID3 response were compared for second and third biologics. Multivariate models controlled for potential confounders.

**Results:**

Of 176 patients whose charts were abstracted, 122 (69.3%) received another anti-TNF agent after they discontinued their first anti-TNF. RAPID3 scores were available for 160 patients. A patient receiving a second bDMARD with another MOA had a higher good or moderate response than a patient receiving anti-TNF (53.5 vs. 30.7%, *p* = 0.01). In the multivariate models, treatment with another MOA was more likely to produce a good RAPID3 response [odds ratio (OR), 2.42; 95% confidence interval (CI), 1.05–5.58] or a good or moderate response (OR, 2.21; 95% CI, 1.23–3.97) than treatment with an anti-TNF.

**Conclusion:**

In patients who have discontinued anti-TNF agents as their first bDMARD, RAPID3 response rates are better for those receiving agents with a different MOA rather than another anti-TNF. Physicians should consider using a bDMARD with a different MOA as the next bDMARD for RA patients whose anti-TNF agent has failed.

**Electronic supplementary material:**

The online version of this article (doi:10.1007/s40744-014-0002-7) contains supplementary material, which is available to authorized users.

## Introduction

The treatment of rheumatoid arthritis (RA) includes biologic disease-modifying anti-rheumatic drugs (bDMARD) that may slow down and control disease progression. Because RA is a chronic disease process, it is essential that patients be periodically assessed for evidence of disease activity or progression [1]. Recurring flares or progressive joint damage may require physicians to consider changes to the patient’s bDMARD regimen. Results from randomized controlled trials indicate that about one-third of RA patients initially treated with anti-tumor necrosis factor α (anti-TNF) agents do not respond, show a suboptimal response, lose response, or develop adverse events [1]. RA patients whose anti-TNF agent as their first bDMARD has failed or who must stop an anti-TNF for some other reason may then switch to another bDMARD, either another anti-TNF agent or a bDMARD with another mechanism of action (MOA).

However, evidence has been mixed [2, 3] regarding the best strategy for subsequent treatments after inadequate response, intolerance, or other reason for discontinuation of an initial anti-TNF agent. Optimal treatment strategies have yet to be defined because no randomized, prospective, head-to-head trials have been conducted comparing the strategy of cycling between TNF inhibitors and using an agent with another MOA [2].

Therefore, an exploratory study was undertaken to compare the response between subsequent use of anti-TNF agents and of bDMARDs with other MOAs in RA patients who have a history of anti-TNF treatment as their first bDMARD.

## Methods

A retrospective chart review study was conducted from February to September 2012 at eight community-based rheumatology practices in the United States (17 investigators). This study was approved by the Western Institutional Review Board and the Copernicus Group Independent Review Board. All procedures followed were in accordance with the ethical standards of the responsible committee on human experimentation (institutional and national) and with the Helsinki Declaration of 1975, as revised in 2000 and 2008. Because of the nature of the study, and as approved by both Institutional Review Boards, informed consent was not obtained.

Patient charts were eligible if the patient’s first bDMARD was an anti-TNF; the patient received a second and/or a third bDMARD between July 1, 2006 and October 1, 2011. They had to be 18 years of age or older at the time of the second bDMARD. bDMARDs included anti-TNF agents (adalimumab, certolizumab, etanercept, golimumab, and infliximab) and bDMARDs with other MOAs (abatacept, rituximab, and tocilizumab).

After study training, site personnel were asked to identify at least 12 charts per participating investigator that met the study eligibility criteria. They were instructed to screen charts starting with the patient who had been most recently seen at the clinic and then to go back chronologically in sequence. Sites performed a one-time data abstraction and entered data into an electronic data collection system. Data elements for abstraction included patient demographics, date of RA diagnosis, laboratory values (cyclic citrullinated peptide and rheumatoid factor), name of bDMARD, and dates of use for up to the first three bDMARDs, whether treatment was ongoing or was discontinued and the reason for discontinuation.

Response was measured by the Routine Assessment of Patient Index Data 3 (RAPID3), which is a composite index of three patient self-report measures: physical function, pain, and patient global assessment of disease activity. The RAPID3 score ranges from 0 to 30, with higher scores indicating more severe disease [4, 5]. RAPID3 provides a feasible approach to monitoring disease activity in the real-world setting. It was used to measure response in this study because it correlates well with other measure of disease activity [4, 5] that may not be routinely available from patient charts.

Unless they had discontinued the bDMARD before the 6-month time point, patients were required to have RAPID3 scores at baseline and at 6 months on treatment for their second or third bDMARD to be eligible for analysis. For the baseline measure, patients had to have a RAPID3 score on the date the biologic was started or up to 6 weeks before the bDMARD start date. Follow-up was defined as 6 months after bDMARD start date, with a window of plus or minus 8 weeks (i.e., 16–32 weeks after the start date).

The 6-month follow-up score was compared with the baseline score to determine RAPID3 response. Good response was defined as a decrease in RAPID3 score of greater than 3.6 points and a follow-up score less than 6. Poor response was defined as a decrease of less than 1.8 points or a decrease between 1.8 and 3.6 points and a follow-up score greater than 12 [6]. In addition, if a patient discontinued therapy before 6 months, the patient was defined as having a poor response. Patients classified as neither good nor poor responders were considered to have had a moderate response.

RAPID3 change scores were calculated by subtracting the baseline score from the 6-month follow-up score. If a patient discontinued therapy before 6 months, the RAPID3 score at the time of discontinuation (plus or minus 4 weeks) was used as the follow-up score. Patients who discontinued but did not have a RAPID3 score available were excluded from this analysis.

The percentages of patients with good RAPID3 response and good or moderate response (i.e., not poor response) were compared between treatments (anti-TNF vs. other MOA) using the Chi square test, and mean change in RAPID3 was compared between treatments using the *t* test. These analyses were stratified by the order of the subsequent bDMARD (i.e., second or third).

Mixed multivariate models were used to account for correlation of multiple observations per patient because data from both second and third biologics were included in the models, if eligible. These models evaluated the effect of treatment (anti-TNF vs. other MOA) on the 6-month RAPID3 outcomes adjusted for potential confounders: age, sex, race/ethnicity, cyclic citrullinated peptide or rheumatoid factor positivity, and baseline RAPID3.

Previous research has suggested that patients who have discontinued one anti-TNF because of lack of efficacy are more likely to discontinue a subsequent anti-TNF agent for the same reason. To assess this occurrence in our study, we included an interaction term for current bDMARD (anti-TNF vs. other MOA) with reason for discontinuation of the previous anti-TNF (lack of efficacy vs. other reason). Lack of efficacy included discontinuation of the anti-TNF because of either primary non-response or secondary non-response (or loss of efficacy after initial response). This subanalysis included all second bDMARD treatments in our data set; third bDMARD treatments were included only if an anti-TNF was the second bDMARD.

All analyses were performed using SAS version 9.2 (SAS Institute Inc., Cary, NC, USA).

## Results

One hundred seventy-six patient charts were abstracted. By study design, all patients received a second bDMARD after discontinuing their first anti-TNF. In addition, 98 patients received a third bDMARD. Treatment sequences are shown in Table 1. Most patients received another anti-TNF after discontinuing the first anti-TNF (*n* = 122; 69.3%). Seventy-nine patients discontinued a second anti-TNF, of these 29 (36.7%) received a third anti-TNF, and 50 received a bDMARD with another MOA.

**Table 1:** bDMARD treatment sequences for 176 patient charts abstracted

Treatment sequence	*N*	%
Anti-TNF → anti-TNF	43	24.4
Anti-TNF → other MOA	35	19.9
Anti-TNF → anti-TNF → anti-TNF	29	16.5
Anti-TNF → anti-TNF → other MOA	50	28.4
Anti-TNF → other MOA → anti-TNF	7	4.0
Anti-TNF → other MOA → other MOA	12	6.8

*Anti*-*TNF* Anti-tumor necrosis factor α, *bDMARD* Biologic disease-modifying anti-rheumatic drug, *MOA* Mechanism of action

The most common reasons for discontinuation of the first anti-TNF were efficacy related (69% discontinued because of lack of initial efficacy or failure to maintain a response). Lack of initial efficacy (primary non-response) was a more common reason for discontinuation as treatment order progressed: 23% of first anti-TNF patients, 40% of second anti-TNF patients, and 48% of third anti-TNF patients discontinued for this reason. In addition, safety/tolerance was reported as a reason for discontinuation more often for anti-TNF as the subsequent biologic (21% for second and 19% for third) than for subsequent bDMARD with other MOA (14% for second and 5% for third).

After excluding treatments when RAPID3 scores were not available for analysis, 215 subsequent bDMARD treatments from 160 patient charts in the analysis of RAPID3 response remained. Of these, 144 were the second bDMARD treatment (101 anti-TNF, 43 other MOA), and 71 were the third bDMARD treatment (29 anti-TNF, 42 other MOA).

Among the 160 patients available for RAPID3 analysis, 121 (75.6%) were female, 139 (86.9%) were white non-Hispanic, and 87 (54.4%) were cyclic citrullinated peptide or rheumatoid factor positive. Mean age at RA diagnosis was 50.0 ± 13.6 years, age at second bDMARD initiation was 56.7 ± 13.0 years, and mean number of years from diagnosis to time of chart review was 9.9 ± 7.2. These characteristics were similar for anti-TNF and other MOA treatments (Table 2).

**Table 2:** Patient characteristics by type of bDMARD

Characteristic	Second biologic			Third biologic		
	Anti-TNF	Other MOA	*P* ^a^	Anti-TNF	Other MOA	*P* ^a^
Patients, *N*	101	43		29	42	
Female, *n* (%)	78 (77.2)	30 (69.8)	0.34	20 (69.0)	34 (81.0)	0.24
White non-Hispanic, *n* (%)	85 (84.2)	41 (95.4)	0.06	25 (86.2)	38 (90.5)	0.58
Age at second DMARD, mean ± SD (years)	56.4 ± 12.6	58.5 ± 14.2	0.38	54.0 ± 10.9	57.3 ± 14.2	0.29
Age at RA diagnosis, mean ± SD (years)	50.3 ± 13.9	50.9 ± 14.2	0.81	48.5 ± 11.4	51.2 ± 14.5	0.40
Number of years since RA diagnosis, mean ± SD	9.2 ± 6.7	10.7 ± 6.5	0.21	9.4 ± 5.5	9.7 ± 9.4	0.89
Cyclic citrullinated peptide or rheumatoid factor positive, *n* (%)	50 (49.5)	28 (65.1)	0.09	15 (51.7)	25 (59.5)	0.51
RAPID3 at baseline, mean ± SD	14.7 ± 6.2	15.2 ± 5.9	0.65	16.0 ± 6.9	18.6 ± 5.4	0.08

^a^ *P* values for categorical variables are from Chi square test and *P* values for continuous variables are from *t* test

*Anti*-*TNF* Anti-tumor necrosis factor α, *bDMARD* Biologic disease-modifying anti-rheumatic drug, *MOA* Mechanism of action, *RA* Rheumatoid arthritis, *RAPID3* Routine Assessment of Patient Index Data 3, *SD* Standard deviation

As shown in Table 2, the RAPID3 score at baseline was similar for patients receiving anti-TNF and other MOA treatments as second bDMARD (14.7 ± 6.2 and 15.2 ± 5.9, respectively; *P* = 0.65). For the third bDMARD, RAPID3 scores were slightly lower in the anti-TNF group than in the other MOA group (16.0 ± 6.9 and 18.6 ± 5.4, respectively; *P* = 0.08), but the difference was not statistically significant.

A greater percentage of patients receiving a bDMARD with other MOA as their second bDMARD had a good RAPID3 response compared with those receiving anti-TNF (Table 3), although this was not statistically significant (18.6 vs. 9.9%, respectively; *P* = 0.15). Patients receiving another MOA as the second bDMARD, however, were more likely to have a good or moderate response (53.5 vs. 30.7%, respectively; *P* = 0.01) and had greater improvement in RAPID3 score (−4.6 ± 5.2 vs. −1.1 ± 5.9, respectively; *P* < 0.01) than patients receiving an anti-TNF. These patterns were similar when analyzing the third bDMARD, but none were statistically significant.

**Table 3:** RAPID3 response at 6 months by type of bDMARD

Characteristic	Second biologic			Third biologic		
	Anti-TNF	Other MOA	*P* ^a^	Anti-TNF	Other MOA	*P* ^a^
Patients, *N*	101	43		29	42	
Good response, *n* (%)	10 (9.9)	8 (18.6)	0.15	4 (13.8)	9 (21.4)	0.41
Good or moderate response, *n* (%)	31 (30.7)	23 (53.5)	0.01	11 (37.9)	21 (50.0)	0.32
RAPID3 change score^b^, mean ± SD	−1.1 ± 5.9	−4.6 ± 5.2	<0.01	−4.1 ± 8.3	−0.6 ± 7.1	0.42
Discontinued <6 months of use, *n* (%)	29 (28.7)	5 (11.6)	0.03	12 (41.4)	9 (21.4)	0.07

*Anti*-*TNF* Anti-tumor necrosis factor α, *bDMARD* Biologic disease-modifying anti-rheumatic drug, *MOA* Mechanism of action, *RAPID3* Routine Assessment of Patient Index Data 3, *SD* Standard deviation

^a^ *P* values for categorical variables are from Chi square test and *P* values for continuous variables are from *t* test

^b^Change score available for *n* = 95 s anti-TNF, *n* = 41 s other MOA, *n* = 25 third anti-TNF, and *n* = 40 third other MOA

One of the criteria for poor response was discontinuation of treatment before 6 months. Patients receiving an anti-TNF as the second bDMARD were more likely to meet this criterion than those receiving another MOA as the second bDMARD (28.7 vs. 11.6%, respectively; *P* = 0.03); though similar trends were observed, this finding was not statistically significant for the third bDMARD (41.4 vs. 21.4%; *P* = 0.07).

After data from subsequent treatments (second and/or third biologic) were included and adjusted for covariates, results of the multivariate mixed models were derived and are shown in Table 4. Treatment with another MOA was more than twice as likely to produce a good RAPID3 response [adjusted odds ratio (OR), 2.42; 95% confidence interval (CI), 1.05–5.58] or a good or moderate response (adjusted OR, 2.21; 95% CI, 1.23–3.97) than treatment with an anti-TNF. In addition, treatment with another MOA resulted in more improvement, estimated as a 2.79 greater decrease in RAPID3 scale (95% CI, −4.54 to −1.05) than treatment with an anti-TNF agent. The interaction term between current bDMARD and reason for discontinuation of previous anti-TNF was not statistically significant for all three outcomes (*P* values ranged from 0.56 to 0.99). bDMARDs with another MOA had an advantage over anti-TNFs regardless of reason for discontinuation. For example, among patients who discontinued their previous anti-TNF because of lack of efficacy, those who switched to a bDMARD with another MOA were 3.33 times more likely to have a good RAPID3 response than those using another anti-TNF, whereas those who discontinued for other reasons were 3.23 times more likely to have a good RAPID3 response (*P* = 0.99 for interaction term).

**Table 4:** Multivariate models of RAPID3 response at 6 months

Outcome	Other MOA vs. anti-TNF OR^a^	Lower 95% CI	Upper 95% CI	*P*
Good response vs. moderate or poor response	2.42	1.05	5.58	0.04
Good or moderate response vs. poor response	2.21	1.23	3.97	0.01

	Regression coefficient^a^	Lower 95% CI	Upper 95% CI	*P*
Change from baseline	−2.79	−4.54	−1.05	<0.01

*Anti*-*TNF* Anti-tumor necrosis factor α, *CI* Confidence interval, *MOA* Mechanism of action, *OR* Odds ratio, *RAPID3* Routine Assessment of Patient Index Data 3

^a^Results adjusted for age, sex, race/ethnicity, cyclic citrullinated peptide or rheumatoid factor positive, and baseline RAPID3

## Discussion

In this exploratory analysis of RA patients who discontinued an anti-TNF agent as their first bDMARD, patients receiving agents with other MOA rather than another anti-TNF as their second or third bDMARD were more than twice as likely to achieve a good or a good or moderate RAPID3 response at 6 months after adjusting for patient demographics, seropositivity, and baseline RAPID3 score. In addition, they showed greater improvement (decrease) in RAPID3 score at 6 months. Although similar patterns were observed with the third bDMARD, statistical significance was not observed. However, patient numbers were smaller and power for analyzing the third bDMARD was lower. Because of these factors, data for the second and third bDMARDs were combined in the multivariate analysis.

Despite having failed an anti-TNF as their first bDMARD, most patients went on to receive another anti-TNF agent as their second bDMARD, and several received another as their third. In a 2006 US survey, 94% of the rheumatologists who responded reported that they switch patients to another anti-TNF agent if the first anti-TNF agent is ineffective or not well tolerated [7]. In the current study, this percentage was 69%, perhaps because more treatment options were available than at the time of the 2006 survey. Even in the current study, however, this treatment pattern represents a large proportion of patients.

Although some studies suggest that a second anti-TNF is effective in patients after the first anti-TNF agent has failed [8], others suggest that response rates for the subsequent anti-TNF agents are progressively lower than for patients using an anti-TNF as their first bDMARD [9–12]. Given the choice of therapeutic options with other MOAs available to patients, the relevant clinical question is whether patients should receive therapy with a different MOA after an anti-TNF agent has failed. Our study is consistent with other studies that have shown that switching to a different MOA might be the more effective strategy.

In several studies of RA patients whose first anti-TNF treatment failed, patients receiving rituximab had better improvement in Disease Activity Score at 28 joints (DAS28) than patients treated with a second anti-TNF [13–16]. A recent Bayesian network meta-analysis [17] of RA patients whose first line anti-TNF failed found that patients receiving bDMARDs with other MOA did better than patients receiving a second anti-TNF agent, as measured by American College of Rheumatology 20 (ACR20) response criteria. Compared with second anti-TNF agents, tocilizumab had an OR of 3.52, rituximab had an OR of 1.87, and abatacept had an OR of 1.64 [17].

A Swiss study found that the benefit of rituximab varied with the reason for switching from a previous TNF inhibitor [15]. Rituximab patients showed greater decrease in DAS28 at 6 months than patients treated with another anti-TNF if the switch occurred because of lack of efficacy. When the switch occurred for another reason, however, the decrease in DAS28 was similar. In the current analysis, we did not find a significant interaction between reason for switching from the previous anti-TNF and response to a biologic with another MOA versus another anti-TNF.

Interpretation of the results should consider the fact that this was a chart review and not a prospective study; thus, data were limited to what was contained in the chart. Because clinical measures of disease activity, such as tender and swollen joint counts, are not routinely recorded in patient charts in clinical practice, we could not assess response based on these outcomes. However, RAPID3 scores have been shown to correlate well with other measures of disease activity [4, 5]. In addition, a few patients (16 of 176) could not be included in the analysis because RAPID3 information was unavailable at key time points. Interpretation should also consider that we examined responses only at 6 months and that longer-term responses were not compared. Finally, the charts were from eight study centers in the United States, which may or may not be representative of all RA patients in generalizing results.

This was an observational study, and patients were not randomly assigned to receive one of the two treatment options. Thus, we could not adjust for any unmeasured or unrecorded factors that might influence treatment selection or impact study outcomes. Nevertheless, available patient characteristics were similar in the two study groups, and we statistically adjusted for any differences by conducting multivariate analyses. Considering the high potential cost of comparing these strategies in a randomized clinical trial, observational studies such as ours can provide input into physicians’ decision-making processes. Although our study protocol included all eight bDMARDs available at the time of the chart review, our final sample sizes were too small to allow for meaningful comparisons between individual bDMARDs (on average, 26 treatments per bDMARD). In other studies, responses were dependent on which bDMARDs were included in the comparisons [18, 19]. We also did not assess the impact of choice of concomitant non-bDMARD on study outcomes. Further research assessing individual treatments in the other MOA group against anti-TNF agents while adjusting for the concomitant non-bDMARD is warranted.

One strength of this analysis is that patients who discontinued treatment were included in the analysis using conservative assumptions. Patients who discontinued bDMARD treatment before 6 months were included in the analysis as poor responders, and their RAPID3 scores at the time of discontinuation, if available, were included for change score calculation. Thus, results were not biased by including only patients who did well on treatment. Our study evaluated the use of an anti-TNF agent compared with another MOA as the second bDMARD and separately as the third bDMARD, which has not been done before this study. In both assessments, the trends were consistent and suggested that patients using other MOAs had better outcomes.

## Conclusion

In conclusion, this study was consistent with previous research in finding that among patients who discontinued anti-TNF agents as their first bDMARD, response rates (as measured by RAPID3 in this study) are better for patients receiving agents with a different MOA rather than another anti-TNF agent. Physicians should consider using a bDMARD with a different MOA as the next bDMARD for RA patients whose treatment with an anti-TNF agent failed. Further research should focus on determining which specific bDMARD with a non-TNF MOA would provide the most benefit to RA patients.

## Electronic supplementary material

Below is the link to the electronic supplementary material.

Supplementary material 1 (PDF 189 kb)
