# Supplementary material for: Response to Biologic Disease-Modifying Anti-Rheumatic Drugs after Discontinuation of Anti-Tumor Necrosis Factor Alpha Agents for Rheumatoid Arthritis
Source: Rheumatol Ther. 2014 Sep 23;1(1):21–30. doi: 10.1007/s40744-014-0002-7 (PMC4883258; doi:10.1007/s40744-014-0002-7)
Supplement: Supplementary file 1 — Supplementary material 1 (PDF 189 kb) [file 40744_2014_2_MOESM1_ESM.pdf]

- A retrospective review of 176 medical charts was conducted at 8 community-based rheumatology practices in the United States in 2012.
- Rheumatoid arthritis patients who had discontinued an anti-tumor necrosis factor α (anti-TNF) agent as their first biologic disease-modifying antirheumatic drugs (bDMARD) were more than twice as likely to achieve a good or a good-or-moderate Routine Assessment of Patient Index Data 3 response at six months when receiving agents with other mechanisms of action rather than another anti-TNF as their second or third bDMARD.
- Despite having failed an anti-TNF as their first biologic DMARD, most patients went on to receive another anti-TNF agent as their second bDMARD and several received another anti-TNF as their third bDMARD.
- The findings of this study may provide physicians with an opportunity to consider bDMARDs other than anti-TNFs to treat patients who have had to discontinue an anti-TNF.

This summary slide represents the opinions of the authors. This study and the article processing charges were sponsored by Genentech, Inc., South San Francisco, California, United States. The authors thank ApotheCom for their editorial support in revising the manuscript during peer review; this support was funded by Genentech, Inc. For a full list of acknowledgments and conflicts of interest for all authors of this article, please see the full text online. Copyright © The Author(s) 2014. Creative Commons Attribution Noncommercial License (CC BY-NC).
